# Primary care patient interest in joining a planned multi‐cancer early detection clinical trial

**DOI:** 10.1002/cam4.7312

**Published:** 2024-05-24

**Authors:** Ronald E. Myers, Mie H. Hallman, Ayako Shimada, Melissa A. DiCarlo, Kaitlyn V. Davis, William T. Leach, Christopher V. Chambers

**Affiliations:** ^1^ Division of Population Science, Department of Medical Oncology Thomas Jefferson University Philadelphia Pennsylvania USA; ^2^ Division of Biostatistics, Department of Pharmacology and Experimental Therapeutics Thomas Jefferson University Philadelphia Pennsylvania USA; ^3^ Department of Family and Community Medicine Thomas Jefferson University Philadelphia Pennsylvania USA

**Keywords:** cancer screening, multi‐cancer detection test, shared decision‐making

## Abstract

**Introduction:**

Clinical trials are being conducted and are being planned to assess the safety and efficacy of multi‐cancer early detection (MCED) tests for use in cancer screening. This study aimed to determine the feasibility of primary care patient outreach in recruiting participants to a planned MCED clinical trial, assess patient interest in trial participation, and measure decisional conflict related to participation.

**Methods:**

The research team used the electronic medical record of a large, urban health care system to identify primary care patients 50–80 years of age who were potentially eligible for a planned MCED trial. We mailed information about the planned MCED trial to identified patients and then contacted the patients by telephone to obtain consent and administer a baseline survey. Subsequently, we contacted consented patients to complete an interview to review the mailed information and elicit perceptions about trial participation. Finally, a research coordinator administered an endpoint telephone survey to assess patient interest in and decisional conflict related to joining the trial.

**Results:**

We randomly identified 1000 eligible patients and were able to make contact with 690 (69%) by telephone. Of the patients contacted, 217 (31%) completed the decision counseling session and 219 (32%) completed the endpoint survey. Among endpoint survey respondents, 177 (81%) expressed interest in joining the MCED trial and 162 (74%) reported low decisional conflict.

**Conclusions:**

Most patients were contacted and about a quarter of those contacted expressed interest in and low decisional conflict about joining the planned MCED trial. Research is needed to determine how to optimize patient outreach and engage patients in shared decision‐making about MCED trial participation.

## 1 INTRODUCTION

Standard of care (SOC) cancer screening testing with periodic mammography (breast), Pap testing (cervical), colonoscopy (colorectal), and low‐dose CT scans (lung) has been shown to decrease mortality and is currently recommended.^1^, ^2^, ^3^, ^4^, ^5^ However, adherence to SOC screening guidelines is suboptimal, there are disparities in screening uptake, and current guidelines relate to a limited number of cancers.^6^

A new generation of multi‐cancer early detection (MCED) tests that evaluate cell‐free DNA (cfDNA) or other biological components is now being developed for use in identifying a wide range of cancers.^7^ However, clinical trials are needed to establish the potential benefits (e.g., reduced cancer‐specific mortality) and harms (e.g., unnecessary procedures and overdiagnosis) of MCED testing.^8^, ^9^, ^10^ As a central component of the Cancer Moonshot^SM^ initiative, the National Cancer Institute aims to support the conduct of clinical trials to determine the safety and efficacy of selected MCED blood tests for detecting cancer and preventing cancer‐related deaths.^11^ The conduct of successful MCED trials will require the identification and engagement of large numbers of participants from diverse populations.^12^, ^13^, ^14^

Primary care practices could provide access to large numbers of patients from diverse populations who are eligible for MCED cancer screening trials. Currently, however, there is a paucity of information on how to identify primary care patients for recruitment to MCED trials and little is known about patient response to an invitation to join an MCED trial.^15^, ^16^, ^17^, ^18^ This study aimed to assess the feasibility of patient outreach to identify and contact primary care patients for a planned MCED clinical trial, engage patients in shared decision making (SDM), determine patient interest in trial participation, and measure decisional conflict related to participation. In terms of secondary aims, we sought to identify factors associated with patient interest in joining the trial.

## 2 MATERIALS AND METHODS

### 2.1 Study design

The research team conducted a prospective, observational study from February 2022 to October 2022 in one internal medicine and three family medicine primary care practices of Jefferson Health, a large, urban health care system. The study was approved by the Jefferson Institutional Review Board (IRB # 21C.806), allowing access to health system electronic medical record (EMR) data to identify cancer screening‐eligible patients for contact.

### 2.2 Participants

Eligible participants included primary care patients who were between 50 and 80 years of age, had no prior diagnosis of cancer, and had a scheduled office visit in the next 2–3 weeks.

### 2.3 Procedures

We identified potential participants through weekly queries of the health system EMR, and attempted telephone contact with each patient to introduce the study, verify eligibility, obtain verbal consent, and administer a baseline survey to enrolled patients. Persons with whom we were able to speak via telephone were defined as “contacted.” The survey collected information on participant sociodemographic characteristics, health literacy, and perceptions of trust in health care researchers. To assess health literacy, we used the Brief Health Literacy Screening Tool (or BRIEF).This tool includes a 4‐item survey with a 5‐point Likert scale.^19^, ^20^ Health literacy categories could range from 4 to 20 (scores of 4–12 = inadequate health literacy, 13–16 = marginal health literacy, and 17–20 = adequate health literacy), with Cronbach's *α* = 0.64.^19^, ^20^

To assess patient trust in health care researchers, we adapted items reported by Shea et al. (2008) on patient perceptions related to health system values and competence and those cited by Mainous et al. (2006) and Hall (2006) that focused on individual beliefs about the honesty of health care researchers and the fidelity with which clinical trial procedures are likely to be applied equitably to persons from diverse backgrounds.^21^, ^22^, ^23^ We adapted eight items from these reports and used a Likert‐type response set (1 = strongly disagree to 5 = strongly agree) for each item (*α* = 0.67). The health research distrust score was calculated as a mean scale score of the eight items with the range of 1–5 (α = 0.67).

Following enrollment, the research team sent each participant a mailing that included a study introduction letter, a copy of the completed verbal consent form, a 1‐page infographic that described the planned MCED trial (included as Supplementary material: Data S1), and a decision factor scoring page. The scoring page was intended for use in a decision counseling telephone call to allow recipients to report the strength and importance of factors that they identified as motivating them to be or not be interested in joining the MCED trial. These materials were developed with input from providers and a panel of patients from the participating practices. In addition, we trained study research coordinators to engage patients in decision counseling by reviewing the patient education materials related to the trial, by didactic training in operating the decision counseling software, and through role play exercises that involved mock interviews.

Seven to 10 days after enrollment, a research coordinator attempted to contact each participant by telephone to complete a decision counseling session. During the session, the research coordinator first reviewed the mailed materials that described the trial and then conducted a semi‐structured interview session using an online software application, the Jefferson Decision Counseling Guide© (or JDCG). The JDCG is a decision aid that can facilitate shared decision‐making (SDM) about a wide range of health care decisions.^24^, ^25^, ^26^, ^27^, ^28^ This tool, which is based on the Analytic Hierarchy Processing framework, is designed to help patients identify decision factors (reasons/goals) that would make them favor one health care option over another.^29^, ^30^, ^31^, ^32^, ^33^, ^34^ A copy of the JDCG is provided (included as Supplementary material: Data S2). During the decision counseling session, the research coordinator asked each participant to identify decision factors that made them favor joining and not joining the planned MCED trial, and entered each elicited decision factor into the decision counseling software application. Then, the research coordinator guided the patient through a structured exercise in which that was designed to elicit how strongly each factor favored one or the other option, as well as their relative importance of each factor to the participant.

Three to 4 weeks after enrollment, a research coordinator attempted to contact each participant again by telephone to conduct an endpoint survey. The survey included 18 items intended to assess respondent perceptions and attitudes toward cancer and the planned MCED trial, anchored in the preventive health model.^35^, ^36^ Each item was scored on a 5‐point Likert scale, and 6 scale scores were computed, and the Cronbach's alpha coefficient was calculated to evaluate the reliability of each scale score: MCED trial salience (4 items, *α* = 0.47); MCED trial response efficacy (2 items, *α* = 0.47); social support (2 items, *α* = 0.46); social influence (2 items, *α* = 0.46); worries and concerns (5 items, *α* = 0.74); and susceptibility to cancer (3 items, *α* = 0.72).

The survey also included four items to assess respondent decisional conflict regarding participation in the planned MCED trial. We used the SURE Scale reported by Légaré et al. (2010) and followed information on using the SURE Scale reported in the Ottawa Decisional Conflict Scale user manual that is available at https://decisionaid.ohri.ca/docs/develop/User_Manuals/UM_decisional_conflict.pdf.^37^ Responses to each item in the SURE Scale were either “Yes” = 1 or “No” = 0, with a summed average for responses summarized as ≤3 = decisional conflict and >3 = no decisional conflict. An overall decisional conflict score was computed (*α* = 0.64).^38^

We also included survey items to assess whether the participant would be interested in participating in the planned MCED trial and whether they wished to be notified upon its initiation. Following a strategy described by Chu et al. for eliciting interest in clinical trial participation, we asked each survey respondent to assume that the trial was open for enrollment and indicate their response to the question “What do you think you would do?”.^39^ The following response options were provided: “I would join,” “I am unsure what I would do,” and “I would not join,” Subsequently, we asked survey respondents if they were interested in being notified when the trial is open, who could answer by indicating either “Yes” or “No.”

Finally, it should be noted that study participants received up to $50 remuneration via ClinCard ($25 for completing the decision counseling session and $25 for completing the endpoint survey).

### 2.4 Data analysis

The study's primary aim was to assess the patient engagement strategy as a method for recruiting primary care patients to MCED trials, and focused on three metrics: (i) the contact rate, that is, the proportion of patients contacted; (ii) the study participation rate (i.e., the proportion of contacted patients who enrolled in the study); and (iii) the decision counseling rate (i.e., the proportion of enrolled patients who completed decision counseling). We estimated these rates, along with 95% confidence intervals (CIs). In addition, we assessed patient interest in joining the planned trial using responses provided on the endpoint survey. We also used endpoint survey responses to measure patient decisional conflict related to trial participation. The research team performed logistic regression analyses to evaluate the associations of baseline survey respondent sociodemographic characteristics, health literacy, and trust in health care researchers with endpoint survey measures of interest in and decisional conflict about joining the MCED trial.

Finally, we used decision counseling interview data entered into the JDCG to identify themes and subthemes related to decision factors related to interest in joining the trial. Specifically, HJ and AI individually coded decision factors and classified them into themes (i.e., cognitive and affective dimensions) and subthemes (i.e., MCED trial salience, MCED trial efficacy, social support/influence and altruism, fears/worries/concerns, and perceived risk and susceptibility). The themes were based on self‐regulation theory, while subthemes mapped constructs of the preventive health model.^35^, ^36^, ^40^, ^41^, ^42^, ^43^ Personal values reflected in the decision factors and their influence related to the options to or not to join the trial were also elicited. Where there was disagreement, REM reviewed the decision factor with the coders and reached agreement on classification. Table S1 displays the guide that our coders used to classify reported decision factors into a cognitive theme and related subthemes (i.e., salience and convenience; efficacy and effectiveness; and social support, influence, and altruism) and an affective theme and related subthemes (i.e., fears, worries and concerns related to potential gains and losses; and perceived risk and susceptibility).

## 3 RESULTS

**FIGURE 1:**
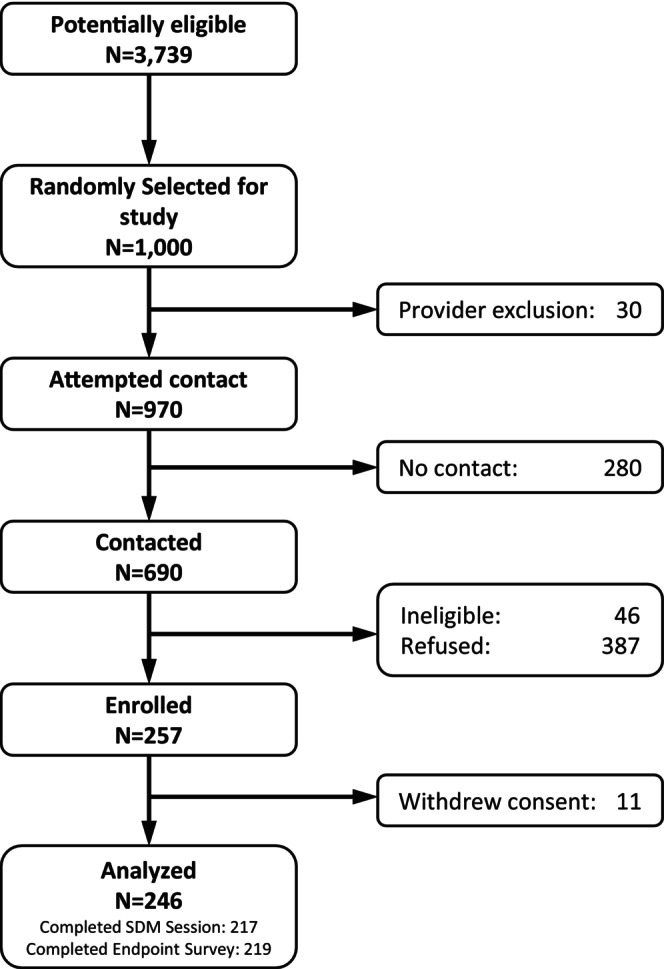
Study design.

Figure 1 shows the steps involved in conducting the study. Between February and October 2022, we identified 3739 unique patients through the EMR who were potentially eligible for the study, and we randomly selected 1000 to approach. We succeeded in contacting 690 of those 1000, for a 69% contact rate, with 95% confidence interval (CI), 66.1%–71.9%. We also enrolled 246 consenting participants, for a 35.7% participation rate (95% CI 32.1%–39.2%). Among study participants, 217 completed the decision counseling session (an 88.2% decision counseling completion rate, 95% CI: 84.2%–92.2%), and 219 completed the endpoint survey (an 89.0% endpoint survey completion rate, 95% CI: 85.1%–92.9%).

Table 1 presents characteristics of those patients who were randomly selected for contact and consented to participate in the research study. Overall, 95% of study participation came from three of the primary care practices, 71% were >60 years of age, 63% were women, 71% were White, 71% had ≥ a high school education, 57% were married, and 52% had a history of smoking. The overwhelming majority had low health research distrust and had high health literacy. Study participation rates were comparable across patient sociodemographic background characteristics.

**TABLE 1:** Characteristics of study participants and nonparticipants.

Characteristic^a^	Participants (*N* = 246)		Nonparticipants (*N* = 754)		Participation rate (row%)
Primary care practice, *n* (%)					
A (internal medicine)	79	(32.1)	160	(21.2)	(33.1)
B (family medicine)	96	(39.0)	381	(50.5)	(20.1)
C (family medicine)	60	(24.4)	165	(21.9)	(26.7)
D (family medicine)	11	(4.5)	48	(6.4)	(18.6)
Age (years), mean (SD)	64.9	(7.9)	64.3	(8.1)	
Age (years), *n* (%)					
50–59	71	(28.9)	248	(32.9)	(22.3)
60–69	95	(38.6)	285	(37.8)	(37.8)
70–80	80	(32.5)	221	(29.3)	(29.3)
Sex, *n* (%)					
Female	155	(63.0)	417	(55.3)	(27.1)
Male	91	(37.0)	337	(44.7)	(21.3)
Race/ethnicity, *n* (%)					
White	174	(70.7)	482	(63.9)	(26.5)
African American	55	(22.4)	176	(23.3)	(23.8)
Hispanic/Latino	5	(2.0)	27	(3.6)	(15.6)
Asian	3	(1.2)	33	(4.4)	(8.3)
Other/unknown	9	(3.7)	36	(4.8)	(20.0)
Marital status, *n* (%)					
Single	52	(21.1)			
Separated/divorced	28	(11.4)			
Widowed	25	(10.2)			
Married	141	(57.3)			
Education, *n* (%)					
High school degree/GED or less	72	(29.3)			
Technical or associate's degree	67	(27.2)			
College graduate and above	107	(43.5)			
Smoking status, *n* (%)					
Never smoker	114	(48.1)	392	(53.1)	(22.5)
Former smoker	82	(34.6)	240	(32.5)	25.5)
Current smoker	41	(17.3)	103	(14.0)	(28.5)
Smoker, NOS/unknown	0	(0.0)	3	(0.4)	(0.0)
Health research distrust, *n* (%)					
Low (1–2.5)	229	(93.5)			
Moderate (2.6–3.5)	15	(6.1)			
High (>3.5)	1	(0.4)			
Health literacy, *n* (%)					
Inadequate (4–12)	13	(5.3)			
Marginal (13–16)	58	(23.7)			
Adequate (17–20)	174	(71.0)			

Abbreviation: NOS, not otherwise specified.

^a^ Information for age, sex, race/ethnicity, and smoking status from the EMR. Information for the remaining variables from the baseline survey, and hence available only for study participants. [Correction added on June 12, 2024 after first online publication. The ‘%’ symbol has been removed throughout from table 1.]

### 3.1 Interest in joining the planned MCED trial and decisional conflict

In total, 177 of the 219 endpoint survey respondents (80.8%, 95% CI: 75.6%–86.0%) reported that they were interested in joining the planned MCED trial; 32 (14.6%) were uncertain about joining the trial; and 10 (4.6%) were not interested. A total of 212 (97%) of the endpoint survey respondents also reported that they wished to be notified, when the trial opened for accrual.

Data presented in Table 2 show that 162 (74%, 95% CI: 68.2%–79.8%) respondents reported no or low decisional conflict related to joining the planned MCED trial. The table also includes information reported in the endpoint survey on patient perceptions about the planned MCED trial. The perceived salience and convenience (e.g., importance) of participating in the trial and anticipated efficacy and effectiveness of the trial scores were very high, while reported fears, worries and concerns, and perceived risk of cancer and susceptibility to negative outcomes related to trial participation scores were low. Perceived social support/influence and altruism were moderate.

**TABLE 2:** Participant perceptions and decisional conflict regarding joining a future MCED trial (*N* = 219).

Perceptions regarding MCED trial participation^a^	Mean	Standard Deviation
Salience	4.7	0.5
Response efficacy	4.6	0.6
Social support	4.3	0.8
Social influence	3.6	1.1
Fears, worries, and concerns	2.4	1.0
Risk and susceptibility	2.1	0.9

Decisional conflict regarding MCED trial participation^b^	*n*	%
0	2	0.9
1	8	3.7
2	20	9.1
3	27	12.3
4	162	74.0

^a^ Each scale score ranges from 1 (low) to 5 (high).

^b^ SURE scale score ranges from 0 (high decisional conflict) to 4 (no decisional conflict).

Tables 3 and 4, respectively, summarize multivariable logistic regression results regarding predictors of interest in joining the planned MCED trial and decisional conflict. Although there was variation across certain characteristics for both outcomes (e.g., race, health care research distrust, and health literacy), none of those characteristics was significantly associated with interest in joining the MCED trial or decisional conflict.

**TABLE 3:** Endpoint survey respondent background characteristics and predictors of interest in joining the planned MCED trial (*N* = 219).

Characteristic	*N* ^b^	Interest in MCED trial participation^a^				
		*n* ^c^	%	OR	95% CI	*p*
Primary care practice						0.799
A (internal medicine)	68	55	80.9	1.00	Ref	
B (family medicine)	82	67	81.7	0.95	0.38, 2.40	0.915
C (family medicine)	58	47	81.0	1.04	0.35, 3.05	0.948
D (family medicine)	11	8	72.7	0.47	0.09, 2.30	0.348
Age (years)						0.166
50–59	61	47	77.0	1.00	Ref	
60–69	86	75	87.2	1.98	0.79, 4.95	0.143
70–80	72	55	76.4	0.87	0.36, 2.09	0.749
Sex						
Female	136	108	79.4	1.00	Ref	
Male	83	69	83.1	1.16	0.53, 2.52	0.718
Race/ethnicity						0.577
White	154	126	81.8	1.00	Ref	
African American	49	37	75.5	0.64	0.23, 1.73	0.376
Other	16	14	87.5	1.36	0.28, 6.68	0.707
Marital status						
Not married	95	78	82.1	1.00	Ref	
Married	124	99	79.8	0.73	0.33, 1.63	0.438
Education						
Less than college	120	97	80.8	1.00	Ref	
College graduate and above	99	80	80.8	1.06	0.47, 2.40	0.894
Smoking status						0.885
Never smoker	100	80	80.0	1.00	Ref	
Former smoker	76	61	80.3	1.14	0.51, 2.58	0.752
Current smoker	35	30	85.7	1.34	0.41, 4.32	0.627
Smoker NOS/unknown	8	6	75.0	0.63	0.11, 3.67	0.603
Health research distrust						
Low (1–2.5)	205	167	81.5	1.00	Ref	
Moderate/high (>2.5)	13	9	69.2	0.53	0.14, 1.96	0.340
Health literacy						0.838
Inadequate (4–12)	12	9	75.0	1.00	Ref	
Marginal (13–16)	50	40	80.0	0.94	0.18, 4.79	0.940
Adequate (17–20)	156	127	81.4	1.20	0.26, 5.47	0.813

*Note*: There was one subject missing responses to both health research distrust and health literacy measures. This subject was excluded from the logistic regression model.

Abbreviations: CI, confidence interval; NOS, not otherwise specified; OR, adjusted odds ratio (model included all the characteristics shown).

^a^ Interest in MCED trial participation: Would join versus unsure or would not join.

^b^ *N* refers to the number of respondents who completed the endpoint survey.

^c^ *n* refers to the number of endpoint survey respondents who expressed interest in joining the trial.

**TABLE 4:** Endpoint survey respondent background characteristics and predictors of decisional conflict related to joining the planned MCED trial (*N* = 219).

Characteristic	*N* ^b^	Decisional conflict about MCED trial participation^a^				
		*n* ^c^	%	OR	95% CI	*p*
Primary care practice						
A (internal medicine)	68	17	25.0	1.00	Ref	
B (family medicine)	82	18	22.0	0.99	0.42, 2.36	0.986
C (family medicine)	58	20	34.5	1.41	0.55, 3.63	0.478
D (family medicine)	11	2	18.2	0.98	0.17, 5.71	0.980
Age (years)						0.613
50–59	61	16	26.2	1.00	Ref	
60–69	86	19	22.1	0.91	0.40, 2.07	0.817
70–80	72	22	30.6	1.35	0.57, 3.16	0.494
Sex						
Female	136	32	23.5	1.00	Ref	
Male	83	25	30.1	1.24	0.61, 2.52	0.554
Race/ethnicity						0.313
White	154	34	22.1	1.00	Ref	
African American	49	18	36.7	1.92	0.76, 4.84	0.166
Other	16	5	31.3	1.80	0.53, 6.12	0.344
Marital status						
Not married	95	24	25.3	1.00	Ref	
Married	124	33	26.6	1.41	0.67, 2.95	0.368
Education						
Less than college	120	28	23.3	1.00	Ref	
College graduate and above	99	29	29.3	1.69	0.78, 3.65	0.182
Smoking status						0.693
Never smoker	100	24	24.0	1.00	Ref	
Former smoker	76	20	26.3	1.15	0.53, 2.48	0.721
Current smoker	35	10	28.6	1.31	0.47, 3.64	0.602
Smoker NOS/unknown	8	3	37.5	2.64	0.51, 13.65	0.246
Health research distrust						
Low (1–2.5)	205	50	24.4	1.00	Ref	
Moderate/high (>2.5)	13	7	53.8	3.21	0.97, 10.60	0.055
Health literacy						0.128
Inadequate (4–12)	12	7	58.3	1.00	Ref	
Marginal (13–16)	50	12	24.0	0.25	0.06, 1.07	0.061
Adequate (17–20)	156	38	24.4	0.25	0.07, 0.98	0.046

*Note*: There was one subject missing responses to both health research distrust and health literacy measures. This subject was excluded from the logistic regression model.

Abbreviations: CI, confidence interval; NOS, not otherwise specified; OR, adjusted odds ratio (model included all the characteristics shown).

^a^ Decisional conflict (Sure) Scale: ≤3 = decisional conflict versus >3 = no decisional conflict.

^b^ *N* refers to the number of respondents who completed the endpoint survey.

^c^ *n* refers to the number of endpoint survey respondents who expressed interest in joining the trial.

Table 5 summarizes the decision factors (*N* = 485) reported by study participants. Each decision factor represents a reason or goal identified by the patient that made them favor either joining the trial or not joining the trial. These factors were categorized by themes and subthemes, using qualitative analysis methods described earlier. Almost three quarters (73%) of the decision factors were cognitive in nature, while the remaining (27%) were affective.

**TABLE 5:** Decision factors by theme, subtheme, and interest in joining the planned trial (*N* = 485).

Theme	Subtheme	Total		Favors to join the MCED trial	
		*N*	%	*n*	%
Total		485		396	82
Cognitive		352	73	292	78
Affective		133	27	104	83
Cognitive	Total	352			
	Salience and convenience	149	42	120	81
	Efficacy and effectiveness	30	9	25	83
	Social support, influence, and altruism	173	49	147	85
Affective	Total	133			
	Fears, worries, and concerns	80	60	58	73
	Risk and susceptibility	53	40	46	87

Cognitive factors mainly reflected social support and altruism (e.g., “to help other people,” or “to contribute to research”) or were related to the perceived salience and convenience of the MCED trial (e.g., “would like to know what is going on with my health” or “potential inconvenience of traveling for blood draw”). Relatively few cognitive factors favoring participation were related to MCED trial efficacy (e.g., “a blood test is a better form of cancer screening”). Affective factors favoring participation included worries and concerns about trial participation and potential findings (e.g., “worried about finding out about cancer” or “concerned about the long‐term time commitment”) and perceived cancer risk and susceptibility (e.g., family history of cancer or having other risk factors for cancer, such as a history of smoking).

There were a small number of cognitive and affective decision factors that favored not joining the planned MCED trial. Those cognitive factors often reflected the belief that participation in the trial would require too much time and effort, while affective factors tended to focus on worry related to the confidentiality of test result information, lack of trust in research, and concern about the potential for being assigned to the control group.

Overall, 82% of the decision factors favored MCED trial participation, and that proportion was similar for cognitive and affective factors (78% and 83%, respectively, Table 5). The majority of factors in all five subthemes favored trial participation, including factors related to fears, worries, and concerns.

## 4 DISCUSSION

Large‐scale clinical trials are needed to evaluate the safety and effectiveness of MCED tests in cancer screening. The conduct of such trials in the context of health system primary care practices would provide access to a substantial number of screening‐eligible patients from diverse populations who could be invited to participate. Work is needed to determine how such a health system‐based approach can be operationalized and the potential impact of operationalizing this type of strategy.

This study assessed the feasibility of an EMR‐based strategy for identifying health system primary care patients who were candidates for a planned MCED clinical trial and steps involved in engaging eligible patients in SDM about trial participation. We found that using EMR data, study personnel were able to contact over two‐thirds of identified patients identified as potentially eligible for the planned trial. Of the patients who were reached, over a third agreed to participate in the current research study, and most study participants expressed interest in joining the trial. McKinney and colleagues, as well as others, have suggested that the need for ancillary support to help clinicians identify patients who are eligible for clinical trials and educate eligible patients about trial procedures.^44^, ^45^, ^46^ Such support is integral not only for ensuring patient engagement and education, but also to facilitate enrollment. Findings reported here highlight the value of persons who can provide ancillary support to clinicians for identifying patients who are eligible for clinical trials and in educating eligible patients about trial procedures as part of the enrollment process. While expressions of interest may not always translate into actual enrollment, it is reasonable to consider expressed interest as an indication of receptivity to trial participation.

We found that interest in joining the planned trial did not differ significantly across patients with different background characteristics. Historically, participation in clinical trials has been lower among racial/ethnic minorities, partly due to lack of awareness or access, distrust in medical research, and limited understanding of clinical trial participation requirements.^47^, ^48^ In our study, there was some indication that patients from racial/ethnic minority backgrounds had a higher level of decisional conflict related to MCED trial participation than Whites, this difference was not statistically significant. Further research is needed to explore the potential effect of race/ ethnicity and health literacy on receptivity to MCED trial enrollment.

The levels of interest and decisional conflict reported by study participants suggest that the primary care setting may offer a setting that is amenable to patient recruitment to MCED trials. It also may be the case that the level of expressed interest in joining an MCED trial may reflect the salutary effect of decision counseling. Patients in other studies who have experienced SDM have reported increased knowledge about health care options, lower decisional conflict related to making a choice, and greater satisfaction with care.^49^, ^50^ As our study's design did not include an assessment of interest before education and decision counseling, the effect of decision counseling is unclear. It is also important to note the positive view of the planned MCED trial and the low level of decisional conflict related to joining the planned trial may not be generalizable. Further research is needed to determine whether integrating a SDM approach into the process of recruiting patients to MCED trials can help address the problem of achieving equity in trial participation.^16^, ^51^, ^52^

Decision factors elicited by study participants were overwhelmingly in favor of MCED trial participation. The decision factors described here reflected reasons/goals that study participants identified as making them favor joining and not joining the trial. As mentioned earlier, most of these factors were cognitive in nature, specifically relating to social support and altruism and the perceived salience or importance of participating in an MCED trial, and concerns related to cancer. These findings are consistent with a prior report that women who perceived participation in a breast cancer biomarker trial to have more benefits than harms and who reported more positive emotions were more likely to decide in favor of participation.^53^

While survey respondents reported relatively few factors that would dissuade them from joining the trial, it is worth noting that some individuals were concerned that trial requirements could be too time‐consuming and that the confidentiality of their medical records data might not be adequately protected. It is important to mention that reported decision factors were related to the planned MCED trial described in the patient infographic. Factors that actually influence decision‐making about joining the trail when the trial is open for recruitment may differ. In future, efforts to engage patients in MCED trials, integrating SDM into the patient recruitment, and consent process may be useful in helping to identify and address such latent barriers to participation.

Overall, study findings indicate that primary care patients may be receptive to MCED trial participation. In another study, our research team found a high level of support for patient participation in such trials among primary care providers.^54^ As suggested earlier, findings reported here suggest efforts to recruit primary care patients to MCED trials may be successful, especially when patient education and decision counseling are integrated into an EMR‐based strategy for identifying eligible patients.^55^, ^56^

Nonetheless, it is important to mention that we asked patients to consider joining a “planned” MCED trial, rather than an ongoing MCED trial that was open at the time of contact. Patient responses might differ when they are offered the opportunity join a trial that is actively recruiting participants and has different requirements. Moreover, patient interest decision conflict levels may change when more complete information about trial participation requirements and possible outcomes are made available. It would also be useful to compare the impact of the decision counseling approach used here with other patient education and engagement strategies. This study recruited patients from four primary care practices affiliated with one urban health system. Most of the patients enrolled in the study were women and were White. Furthermore, most participants had a very low level of health research distrust and high health literacy. Thus, the generalizability of study findings may be limited.

## AUTHOR CONTRIBUTIONS

**Ronald E. Myers:** Conceptualization (equal); funding acquisition (equal); investigation (equal); resources (equal); supervision (equal); writing – original draft (equal); writing – review and editing (equal). **Mie H. Hallman:** Data curation (equal); formal analysis (equal); investigation (equal); project administration (equal); writing – review and editing (equal). **Ayako Shimada:** Data curation (equal); formal analysis (equal); writing – review and editing (equal). **Melissa A. DiCarlo:** Conceptualization (equal); investigation (equal); project administration (equal); writing – review and editing (equal). **Kaitlyn V. Davis:** Investigation (equal); writing – original draft (equal); writing – review and editing (equal). **William T. Leach:** Data curation (equal); formal analysis (equal); writing – original draft (equal); writing – review and editing (equal). **Christopher V. Chambers:** Conceptualization (equal); funding acquisition (equal); investigation (equal); resources (equal); supervision (equal); writing – original draft (equal); writing – review and editing (equal).

## FUNDING INFORMATION

This work was supported by a grant from Exact Sciences Corporation, Madison, WI, as well as by Thomas Jefferson University's Sidney Kimmel Cancer Center [Cancer Center Support Grant 5P30CA056036‐17].

## IRB STATEMENT

The study was approved by the Jefferson Institutional Review Board (IRB # 21C.806) and all participants provided verbal informed consent.

## Supporting information


Table S1.

Data S1.

Data S2.

## Data Availability

Data available upon request.
