# Correction to “primary care patient interest in joining a planned multi‐cancer early detection clinical trial”

**DOI:** 10.1002/cam4.7427

**Published:** 2024-06-25

Myers RE, Hallman MH, Shimada A, et al. primary care patient interest in joining a planned multi‐cancer early detection clinical trial. *Cancer Med*. 2024; 13:e7312. doi:10.1002/cam4.7312.

The word, “Interests” in the title should be “Interest.”

In Table 1, the “%” symbol in the parentheses in columns 2 and 4 was removed, as they were redundant.

We apologize for these errors.

