# Supplementary material for: Primary care patient interest in joining a planned multi‐cancer early detection clinical trial
Source: Cancer Med. 2024 May 24;13(10):e7312. doi: 10.1002/cam4.7312 (PMC11117448; doi:10.1002/cam4.7312)
Supplement: Supplementary file 1 — Table S1. [file CAM4-13-e7312-s003.doc]

**Supplemental Table 1. Guide for Coding Decision Factors and Preference**

**Related to Joining a Clinical Trial**

**Theme Subtheme Decision Factor Preference**

**Cognitive Salience & Coherence**

Important To join

Not important Not to join

Easy/convenient To join

Difficult/inconvenient Not to join

**Cognitive Efficacy & Effectiveness**

Likely to be successful To join

Not likely to be successful Not to join

**Cognitive Social Support, Influence & Altruism**

Recommended/supported To join Not recommended/Not supported Not to join Trust in research To join

Do not trust in research Not to join

**Affective Fears, Worries & Concerns**

Feel fear/worry/concern To join

Do not feel fear/worry/concern Not to join

**Affective Perceived Susceptibility**

Feel need to address risk

Age To join

Family history To join

Personal history To join

Past exposure To join Do not feel need to address risk

Age Not to join

Family history Not to join

Personal history Not to join

Past exposure Not to join
