# Supplementary figures and images for: Primary care patient interest in joining a planned multi‐cancer early detection clinical trial
Source: Cancer Med. 2024 May 24;13(10):e7312. doi: 10.1002/cam4.7312 (PMC11117448; doi:10.1002/cam4.7312)

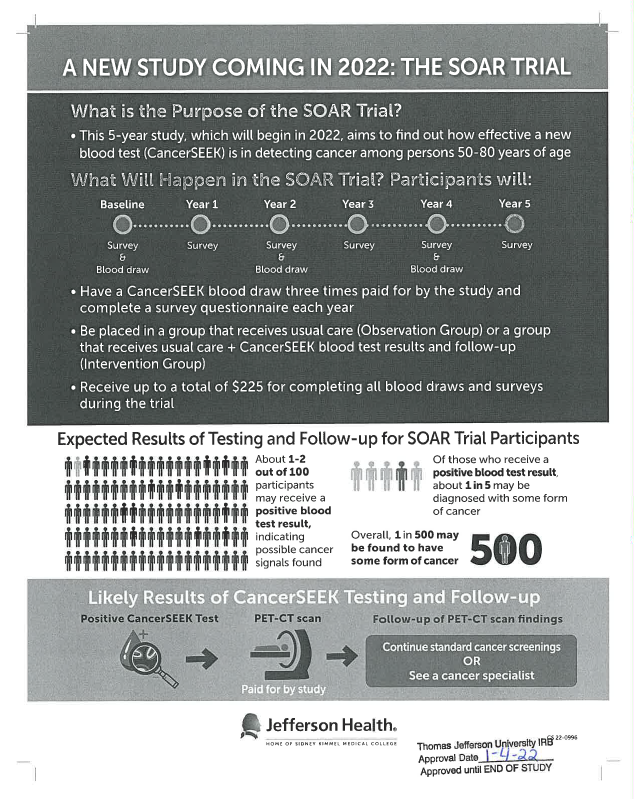

Supplement: Supplementary file 2 — Data S1. [file CAM4-13-e7312-s001.docx]
