# Supplementary material for: Primary care patient interest in joining a planned multi‐cancer early detection clinical trial
Source: Cancer Med. 2024 May 24;13(10):e7312. doi: 10.1002/cam4.7312 (PMC11117448; doi:10.1002/cam4.7312)
Supplement: Supplementary file 3 — Data S2. [file CAM4-13-e7312-s002.doc]

**Decision Counseling Guide**

**Decision to Be Made:**

Patient:

MRN:

Counselor:

ID:

Date:

To/Not to Join the MCED trial

Patients have values that may favor **Option A:**

To join the trial

or **Option B:** Not to join the trial

Use this guide to identify those values (reasons/goals), weigh their effects and importance, and clarify preference.

**STEP ONE**: Help the patient identify reasons/goals that make them favor one option (A/B) over the other (A/B), select the most important reasons/goals (up to the top 3), and rank those in order of importance (1= most important, 2 = 2nd most important, 3 = 3rd most important). Enter the top reason(s)/goal(s) in **STEP TWO**.

# Reason(s)/Goal(s) Favoring Option A over B Reason(s)/Goal(s) Favoring Option B over A

**STEP TWO**: Review the top reason(s)/goal(s) with the patient. Then, ask how much more each one makes the patient favor the related Option (A/B) over the other Option (A/B). Example: "*It seems that 1. Getting peace of mind makes you favor joining the trial more than not joining the trial How much more?*" If there is one reason/ goal, complete this step. If there are two or three reasons/goals, complete this step for those reasons/goals and proceed to **STEP THREE.**

# Level of Effect

| About | A | Some- |  | Very |  |
| --- | --- | --- | --- | --- | --- |
| the | Little | what | Much | Much | Overwhelmingly |

**Top Reason(s)/Goal(s)**


# Same More More More More More


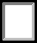

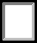

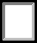

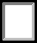

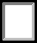

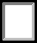

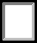

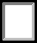

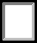

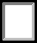

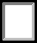

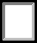

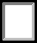

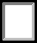

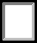

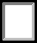

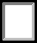

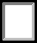


**STEP THREE**: Ask the patient how much more important one reason/goal is than the other reason/goal. Example: "*How much more important is 1. Getting peace of mind than 2. Avoiding bad news?*" If there are two reasons/goals, enter how much more important 1 is compared to 2. If there are three reasons/goals, enter the importance of 1 compared to 2, 2 compared to 3, and 1 compared to 3.

# Level of Importance

| About | A | Some- |  | Very |  |
| --- | --- | --- | --- | --- | --- |
| the | Little | what | Much | Much | Overwhelmingly |
| Same | More | More | More | More | More |

**Top Reason(s)/Goal(s)**

1. compared to **2**.


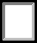

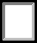

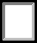

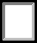

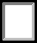

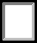

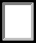

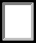

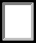

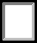

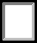

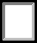

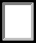

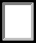

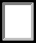

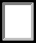

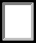

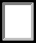


1. compared to **3**.
2. compared to **3**.
